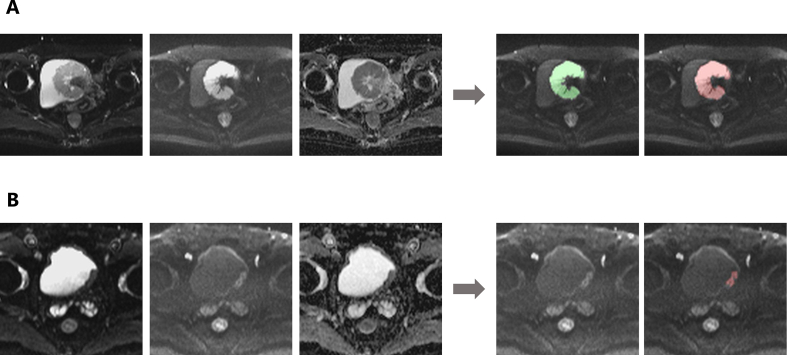# Development of deep learning model for diagnosing muscle-invasive bladder cancer on MRI with vision transformer

**DOI:** 10.1016/j.heliyon.2024.e36144

**Published:** 2024-08-10

**Authors:** Yasuhisa Kurata, Mizuho Nishio, Yusaku Moribata, Satoshi Otani, Yuki Himoto, Satoru Takahashi, Jiro Kusakabe, Ryota Okura, Marina Shimizu, Keisuke Hidaka, Naoko Nishio, Akihiko Furuta, Aki Kido, Kimihiko Masui, Hiroyuki Onishi, Takehiko Segawa, Takashi Kobayashi, Yuji Nakamoto

**Affiliations:** a Department of Diagnostic Imaging and Nuclear Medicine, Graduate School of Medicine, Kyoto University, 54, Shogoin Kawahara-cho, Sakyo-Ku, Kyoto, 606-8507, Japan; b Department of Radiology, Shiga General Hospital, 4-30, Moriyama 5-chome, Moriyama-shi, Shiga, 524-8524, Japan; c Department of Radiology, Kyoto City Hospital, 2-1 Mibu Higashi Takada-cho Nakagyo-ku, Kyoto, 604-8845, Japan; d Department of Radiology, Takatsuki General Hospital, 1-3-13, Kosobe-Cho, Takatsuki-Shi, Osaka, 569-1192, Japan; e Department of General Surgery, Cleveland Clinic, Cleveland, OH, USA; f Department of Radiology, Osaka Red Cross Hospital, 5-30 Fudegasakicho, Tennoji-ku, Osaka, 543-0027, Japan; g Department of Urology, Kyoto University Hospital, 54 Shogoin Kawahara-cho, Sakyo-Ku, Kyoto, 606-8507, Japan; h Department of Urology, Osaka Red Cross Hospital, 5-30 Fudegasakicho, Tennoji-ku, Osaka, 543-0027, Japan; i Department of Urology, Kyoto City Hospital, 2-1 Mibu Higashi Takada-cho Nakagyo-ku, Kyoto, 604-8845, Japan

**Keywords:** Bladder cancer, Deep learning, Convolutional neural network, MRI, Vision transformer

## Abstract

**Rationale and objectives:**

To develop and validate a deep learning (DL) model to automatically diagnose muscle-invasive bladder cancer (MIBC) on MRI with Vision Transformer (ViT).

**Materials and methods:**

This multicenter retrospective study included patients with BC who reported to two institutions between January 2016 and June 2020 (training dataset) and a third institution between May 2017 and May 2022 (test dataset). The diagnostic model for MIBC and the segmentation model for BC on MRI were developed using the training dataset with 5-fold cross-validation. ViT- and convolutional neural network (CNN)-based diagnostic models were developed and compared for diagnostic performance using the area under the curve (AUC). The performance of the diagnostic model with manual and auto-generated regions of interest (ROI_manual_ and ROI_auto_, respectively) was validated on the test dataset and compared to that of radiologists (three senior and three junior radiologists) using Vesical Imaging Reporting and Data System scoring.

**Results:**

The training and test datasets included 170 and 53 patients, respectively. Mean AUC of the top 10 ViT-based models with 5-fold cross-validation outperformed those of the CNN-based models (0.831 ± 0.003 vs. 0.713 ± 0.007–0.812 ± 0.006, p < .001). The diagnostic model with ROI_manual_ achieved AUC of 0.872 (95 % CI: 0.777, 0.968), which was comparable to that of junior radiologists (AUC = 0.862, 0.873, and 0.930). Semi-automated diagnosis with the diagnostic model with ROI_auto_ achieved AUC of 0.815 (95 % CI: 0.696, 0.935).

**Conclusion:**

The DL model effectively diagnosed MIBC. The ViT-based model outperformed CNN-based models, highlighting its utility in medical image analysis.

## Abbreviations

AUC =: Area under the curve
BC =: Bladder cancer
CNN =: Convolutional neural network
DWI =: Diffusion-weighted image
NMIBC =: Non-Muscle-Invasive Bladder cancer
MIBC =: Muscle-invasive bladder cancer
TURBT =: Transurethral resection of bladder tumor
T2WI =: T2-weighted image
VI-RADS =: Vesical Imaging Reporting and Data System
ViT =: Vision Transformer

## 1 Introduction

Bladder cancer (BC) is one of the most common malignancies globally and the sixth most common cancer in men [1]. The T stage, which indicates whether a tumor is non-muscle-invasive (T1 or lower) or muscle-invasive (T2 or higher), is important for determining the treatment strategy. The gold standard for differentiating non-muscle-invasive BC (NMIBC) from muscle-invasive BC (MIBC) is the pathological analysis of the sample obtained via transurethral resection of the bladder tumor (TURBT). However, the quality of samples obtained via TURBT often varies with the urologist. Additionally, a single TURBT may underdiagnose muscle invasion by 20–30 % [2,3]. Therefore, MRI plays an important role in the preoperative evaluation of BC in clinical practice [4,5].

Vesical Imaging-Reporting and Data System (VI-RADS) is a standard imaging and diagnostic method for BC [6]. Although there are previous reports including radiomics research on its usefulness, substantial effort is required for in assessing BC, considering that BC is often multiple [7]. Moreover, inter-reader agreement varied in previous reports [[8], [9], [10]]. Therefore, an objective, highly accurate, and automated diagnostic model for diagnosing MIBC is warranted.

Deep learning is highly advantageous in medical imaging and analysis because it can automatically extract and learn useful features from images, leading to improved performance and accuracy in various tasks [11]. A few recent studies have reported convolutional neural networks (CNN)-based deep learning models for predicting MIBC on MRI. However, they did not adequately assess the generalization of the models [[12], [13], [14]]. In addition, these studies require manual segmentation of the tumor and bladder or the entire tumor, which could be a critical barrier to clinical application.

Vision Transformer (ViT) is a deep learning model that has revolutionized image analysis by employing self-attention mechanisms. CNN, which has been the mainstream methods in medical image analysis, extracts local patterns and features using convolutional layers, while ViT splits the entire image into small patches and learns their relationships at early layers; therefore, it is better than CNN for learning overall image relationship [15,16]. ViT achieves this by splitting the entire image into small patches and learning their relationships at early layers [15]. ViT outperforms CNNs on various tasks and has achieved state-of-the-art performance with several reports of its application in medical image analysis [17].

Therefore, in this study, we aimed to develop and validate a clinically useful deep-learning model that automatically diagnoses MIBC using MRI, and to compare the diagnostic performance of ViT and CNN-based models.

## 2 Materials and Methods

This multicenter retrospective study adhered to the principles of the Declaration of Helsinki and the Standards for Reporting of Diagnostic Accuracy Studies guidelines [18]. Ethical approval was obtained from the institutional review boards of Kyoto University Hospital (Institution 1) (R2695-1), Japanese Red Cross Osaka Hospital (Institution 2) (J-0202), and Kyoto City Hospital (Institution 3) (R714). The requirement for informed consent was waived owing to the retrospective study design.

### 2.1 Datasets

The training dataset included the data of patients pathologically diagnosed with BC who underwent preoperative MRI at institutions 1 and 2 between January 2016 and June 2020. They had participated in a previous study on BC segmentation [19]. The external test dataset included the data of participants who underwent preoperative MRI at institution 3 between May 2017 and May 2022. Muscle invasion was determined using the patient clinical and pathological records and according to the European Association of Urology guidelines [20]. This guideline recommends second TURBT for patients diagnosed with Ta or T1 on initial TURBT who are at high risk for residual tumor. The exclusion criteria were (a) prior treatment with TURBT or intravesical therapy within 6 months, (b) uncertain T-stage, (c) insufficient MRI sequences, (d) severe artifacts, and (e) no detectable BC on MRI. The final training dataset comprised 170 (institution 1, n = 84; institution 2, n = 86) out of the initial 322 patients with BC (institution 1, n = 168; institution 2, n = 154). The external test dataset consisted of the data of 53 of the 70 initially identified patients with BC (Fig. 1). The MRI acquisition and image preprocessing parameters are detailed in Appendices A and B and Table S1. Three-sequence MR images (Diffusion-weighted image [DWI] of b = 0 and 800 or 1000 s/mm^2^ and apparent diffusion coefficient [ADC] map) with the largest cross-section of the BC were used as input data, because DWI is the most useful sequence for diagnosing MIBC and considered the dominant sequence on VI-RADS [6]. Fig. 2 presents an overview of the study.

**Fig. 1:**
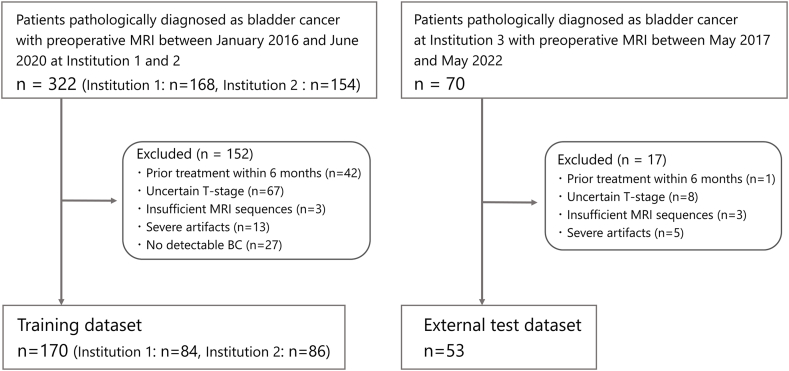
Flowchart of patient inclusion for the study.

**Fig. 2:**
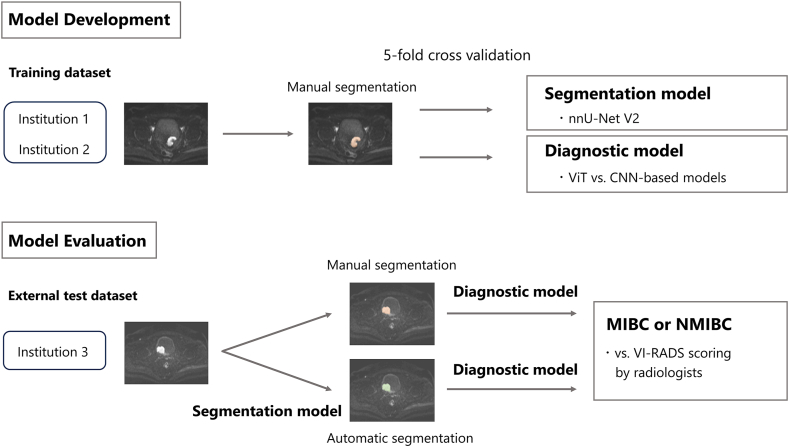
Overview of the study. We developed a deep learning model to predict muscle invasion in bladder cancer (BC) using MRI. First, we developed a segmentation model for automated segmentation of BC on MR images and a diagnosis model to detect MIBC using a training dataset with 5-fold cross-validation. The training dataset consisted of the data of patients with BC from two institutions; the BCs were manually segmented using MRI. We compared the diagnostic performances of the ViT- and CNN-based models. Second, the model was evaluated using an external test dataset. We created input data using manual and automated (using the developed model) segmentation of the BC. For each input data point, the diagnostic model predicted whether the tumor was MIBC or NMIBC. The diagnostic performance of the model was compared with that of radiologists using VI-RADS scoring. BC: Bladder cancer; ViT: Vision Transformer; CNN: Convolutional neural network; MIBC: Muscle-invasive bladder cancer; NMIBC: Non-muscle invasive bladder cancer; and VI-RADS: Vesical Imaging Reporting and Data System.

### 2.2 Tumor segmentation

The BCs were manually segmented on MRI using 3D Slicer (version 5.2.2) (https://www.slicer.org). For multiple BCs, the largest tumor was segmented. Two board-certified radiologists specializing in urogenital radiology determined the regions of interest (ROIs) by consensus: Y.M. and Y.K. (13-year experience each) for the training dataset and S.O. and Y.K. (11- and 13-year experiences, respectively) for the external test dataset.

### 2.3 Data partition

The training dataset was randomly split into five datasets on patient basis for developing the model with five-fold cross-validation. The external test dataset was only used to assess the diagnostic performance of the final model.

### 2.4 Model development

#### 2.4.1 Segmentation model

A segmentation model based was developed on the manually segmented ROIs for the training dataset using nnU-Net. U-Net is a promising fully CNN architecture for the segmentation of medical images, including those of BC, and nnU-Net is a U-Net-based semantic segmentation algorithm that automatically adapts to a given dataset [[19], [21], [22]]. The input data were 3D three-sequence images (DWI of b = 0 and 800 or 1000 s/mm^2^ and ADC map). The default configurations of nnU-Net V2 (3D U-Net) were used for model training. For the external test dataset, the ROIs were generated manually and automatically using this model.

#### 2.4.2 Diagnostic model

A diagnostic model was developed based on ViT (ViT-L/16) pre-trained on ImageNet using the PyTorch framework (Figure S1) [15,23]. Five-fold cross-validation was applied during training; the hyperparameters are provided in Appendix C. For comparison, 5-fold cross-validation was performed using 10 representative CNN-based models with the same hyperparameters as the ViT-based model: VGG16, ResNet34, ResNet50, ResNet101, DenseNet121, and EfficientNet (B0, B1, B2, B3, and B4) [[24], [25], [26], [27]]. The mean AUCs of the top 10 of the 40 sets of models during cross-validation were compared. The source code for our ViT-based model is available at https://github.com/YasKurata/BC_ViT.

### 2.5 Model evaluation

The ViT-based model with the lowest mean loss for the 5-fold cross-validation was adopted as the final model. To evaluate the final model using the external test dataset, an ensemble method was used with five models developed by 5-fold cross-validation.

### 2.6 VI-RADS scoring by radiologists

Three experienced urogenital radiologists (R1, R2, and R3) with 14, 30, and 15 years of experience, respectively, and three junior radiologists (R4, R5, and R6) with three, four, and three years of experience in radiology, respectively, assessed the bladder MRI of the external test dataset using VI-RADS criteria [6]. The radiologists referred to all the available slices and sequences including T1 and T2-weighted image (T2WI) and DWI. Prior to the evaluation, all the readers were educated about the VI-RADS criteria through 10 practice cases that were not part of the external test dataset.

### 2.7 Statistical analysis

The statistical analyses were performed using JMP Pro (version 16; SAS Institute Inc.) and EZR (version 1.61; Saitama Medical Center, Jichi Medical University, Saitama, Japan) [28]. The patient characteristics were compared using t-tests for continuous variables and Fisher's exact tests for categorical variables. The models were evaluated for sensitivity, specificity, accuracy, positive and negative predictive values, and area under the curve (AUC) to diagnose MIBC. The Youden index was used to set the cutoffs. The mean AUCs of the diagnostic models were compared using *t*-test. Interobserver agreement was calculated using weighted Cohen's kappa statistics, with values of 0.21–0.40, 0.41–0.60, 0.61–0.80, and 0.81–1.00 representing fair, moderate, substantial, and excellent agreement, respectively [29]. P < .05 denoted statistical significance.

## 3 Results

### 3.1 Patient characteristics

The patient characteristics are presented in Table 1. The training dataset consisted of the data of 170 patients (mean age, 73.6 ± 9.0; 136 males and 34 females), and the external test dataset included the data of 53 patients (mean age, 73.2 ± 10.3; 40 males and 13 females). In total, 62 of 170 patients in the training dataset and 22 of 53 patients in the external test dataset had MIBC. No statistically significant differences were observed in the clinical characteristics between the training and external test datasets.

**Table 1:** Patient characteristics.

	Training dataset	Test dataset	p-value
Number of patients	170	53	
Age (years, mean ± SD)	73.6 ± 9.0	73.2 ± 10.3	0.81
Sex			0.56
Male	136 (80)	40 (75)	
Female	34 (20)	13 (25)	
Pathological T stage			0.74
Ta	59 (35)	17 (32)	
T1	49 (29)	14 (26)	
T2	36 (21)	14 (26)	
T3	21 (12)	8 (15)	
T4	5 (3)	0 (0)	
Histological grade			0.58
High	136 (80)	46 (87)	
Low	30 (18)	6 (11)	
Others	4 (2)	1 (2)	
Muscle invasion			0.51
MIBC	62 (36)	22 (42)	
NMIBC	108 (64)	31 (58)	

Unless otherwise stated, data are presented as the number of patients with percentages in parentheses.

SD: standard deviation.

MIBC: muscle-invasive bladder cancer.

NMIBC: non-muscle invasive bladder cancer.

### 3.2 Model development

The mean AUCs for the 5-fold cross-validation of the top 10 ViT- and CNN-based models on the training dataset are listed in Table 2. Of the CNN-based models, the ResNet34 and EfficientNet had the lowest (0.713 ± 0.007) and highest (0.812 ± 0.006) mean AUCs, respectively. These results indicate that larger models do not always demonstrate better diagnostic performance. The ViT-based model significantly outperformed other CNN-based models (p < .001 for all the CNN-based models). The mean AUC of the final model for the 5-fold cross-validation was 0.862 ± 0.084 (Appendix C).

**Table 2:** Mean AUC of the top 10 ViT and CNN-based models for 5-fold cross-validation on the training dataset.

Base model	Mean AUC
ViT	0.831 ± 0.003
Vgg16	0.765 ± 0.004
ResNet34	0.713 ± 0.007
ResNet50	0.732 ± 0.004
ResNet101	0.727 ± 0.007
Densenet121	0.725 ± 0.004
EfficientNet B0	0.758 ± 0.010
EfficientNet B1	0.771 ± 0.006
EfficientNet B2	0.794 ± 0.005
EfficientNet B3	0.741 ± 0.005
EfficientNet B4	0.812 ± 0.006

Data are presented as mean ± standard deviation. The mean AUC of the ViT-based model was significantly higher than that of all CNN-based models (p < .001).

AUC: Area under the curve.

ViT: Vision transformer.

CNN: Convolutional neural network.

### 3.3 Model evaluation

**Fig. 3:**
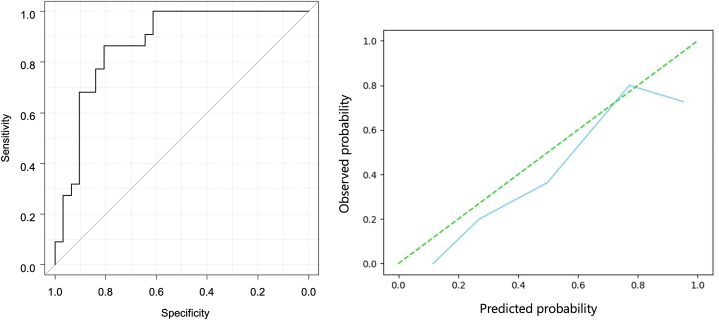
Receiver operating characteristic (ROC) curve and calibration plot for the Vision Transformer-based diagnosis model for the external test dataset.

The ViT-based final model revealed AUC, sensitivity, specificity, accuracy, positive predictive value, and negative predictive values of 0.872 (95 % confidence interval [CI]:0.777, 0.968), 0.864 (19/22) (95 % CI: 0.0651, 0.971), 0.806 (25/31) (95 % CI:0.625, 0.925), 0.830 (44/53) (95 % CI:0.702, 0.919), 0.760 (19/25) (95 % CI:0.549, 0.906), and 0.893 (25/28) (95 % CI:0.718, 0.977), respectively, for the external test dataset. Fig. 3 shows the receiver operating characteristic (ROC) curve and calibration plot. Table S2a presents the confusion matrix for the final model.

### 3.4 Diagnostic performance of radiologists

**Fig. 4:**
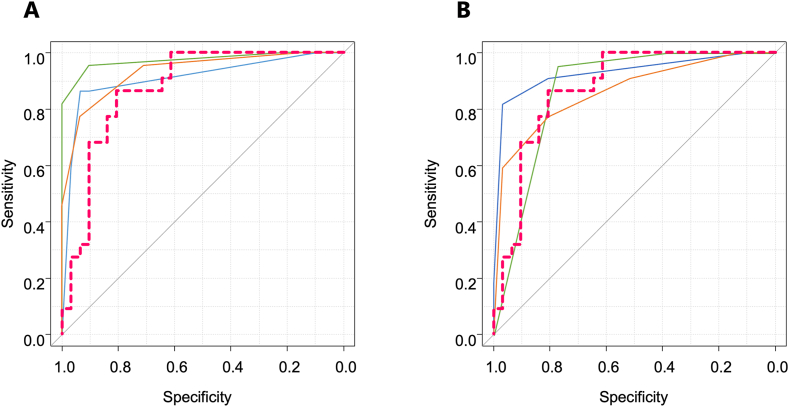
Receiver operating characteristic (ROC) curves for the radiologists. (A) ROC curves of R1 (blue line), R2 (green line), R3 (orange line), and the diagnosis model (red dotted line). (B) ROC curves of R4 (blue line), R5 (green line), R6 (orange line), and the diagnosis model (red dotted line). (For interpretation of the references to colour in this figure legend, the reader is referred to the Web version of this article.)

**Fig. 5:**
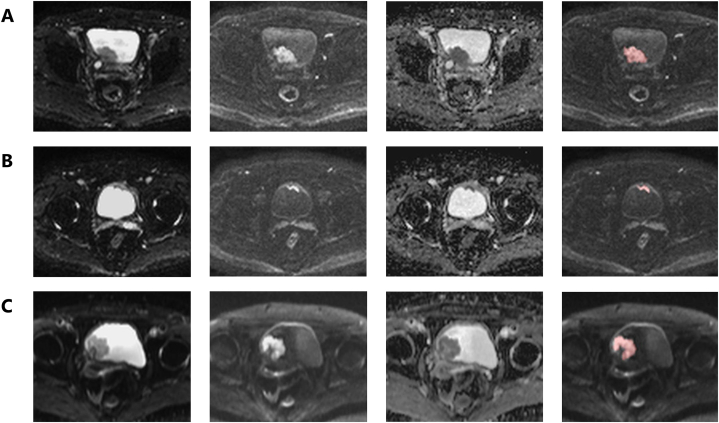
Three representative cases of bladder cancer (BC) in the external test dataset. Left to right: Diffusion-weighted images (b = 0 s/mm^2^), diffusion-weighted images (b = 1000 s/mm^2^), apparent diffusion coefficient map, and manually segmented region of interest in the BC. (A) Male patient in his 60s with MIBC. Pelvic MRI revealed an exophytic tumor without a stalk on the posterior bladder wall. All six radiologists assigned a VI-RADS score of 4. The output of the ViT-based diagnostic model was 0.976, which is a strong indicator of MIBC. (B) Male patient in his 60s with NMIBC. Pelvic MRI revealed a slightly exophytic tumor without a stalk on the anterior wall of the bladder. Four radiologists assigned a VI-RADS score of 4, and the other two radiologists assigned a VI-RADS score of 3. The output of the ViT-based diagnostic model was 0.301, which is indicative of NMIBC. (C) Female patient in her 70s with NMIBC. Pelvic MRI revealed an exophytic tumor with a stalk on the right lateral wall of the bladder. All six radiologists assigned a VI-RADS score of 2. The output of the ViT-based diagnostic model was 0.515, which was more suggestive of MIBC. BC: Bladder cancer; MIBC: Muscle-invasive bladder cancer; NMIBC: Non-muscle invasive bladder cancer; VI-RADS: Vesical Imaging Reporting and Data System; and ViT: Vision Transformer.

The diagnostic performances of the radiologists for the external test dataset with different thresholds (VI-RADS ≥3 and VI-RADS ≥4) are presented in Table 3. The AUCs of the experienced radiologists were 0.909 (95 % CI: 0,827, 0.991), 0.973 (95 % CI: 0.931, 1.00), and 0.931 (95 % CI: 0.866, 0.966). The AUCs of the junior radiologists were 0.930 (95 % CI: 0.857, 1.00), 0.873 (95 % CI: 0.780, 0.967), and 0.862 (95 % CI: 0.761, 0.963). The ROC curves for the VI-RADS scores of the radiologists are shown in Fig. 4. The interobserver agreement of the experienced radiologists was excellent (κ = 0.81−0.90), whereas that of the junior radiologists was substantial (κ = 0.68−0.78). The overall interobserver agreement was substantial to excellent (κ = 0.61–0.90) (Table S3). Representative BC cases from the external test dataset are shown in Fig. 5.

**Table 3:** Diagnostic performance of the radiologists and diagnostic model for the external test dataset.

Reader	Sensitivity	Specificity	Accuracy	PPV	NPV	AUC
R1						0.909 (0.827, 0.991)
VI-RADS ≥3	86.4 [19/22] (65.1, 97.1)	90.3 [28/31] (74.2, 98.0)	88.7 [47/53] (77.0, 95.7)	86.4 [19/22] (65.1, 97.1)	90.3 [28/31] (74.2, 98.0)	
VI-RADS ≥4	86.4 [19/22] (65.1, 97.1)	93.5 [29/31] (78.6, 99.2)	90.6 [48/53] (79.3, 96.9)	90.5 [19/21] (69.6, 98.8)	90.6 [29/32] (75.0, 98.0)	
R2						0.973 (0.931, 1.00)
VI-RADS ≥3	95.5 [21/22] (77.2, 99.9)	90.3 [28/31] (74.2, 98.0)	92.5 [49/53] (81.8, 97.9)	87.5 [21/24] (67.6, 97.3)	96.6 [28/29] (82.2, 99.9)	
VI-RADS ≥4	81.8 [18/22] (59.7, 94.8)	100.0 [31/31] (83.8, 100.0)	92.5 [49/53] (81.8, 97.9)	100.0 [18/18] (74.0, 100.0)	88.6 [31/35] (73.3, 96.8)	
R3						0.931 (0.866, 0.996)
VI-RADS ≥3	95.5 [21/22] (77.2, 99.9)	71.0 [22/31] (52.0, 85.8)	81.1 [43/53] (68.0, 90.6)	70.0 [21/30] (50.6, 85.3)	95.7 [22/23] (78.1, 99.9)	
VI-RADS ≥4	77.3 [17/22] (54.6, 92.2)	93.5 [29/31] (78.6, 99.2)	86.8 [46/53] (74.7, 94.5)	89.5 [17/19] (66.9, 98.7)	85.3 [29/34] (68.9, 95.0)	
R4						0.930 (0.857, 1.00)
VI-RADS ≥3	90.9 [20/22] (70.8, 98.9)	80.6 [25/31] (62.5, 92.5)	84.9 [45/53] (72.4, 93.3)	76.9 [20/26] (56.4, 91.0)	92.6 [25/27] (75.7, 99.1)	
VI-RADS ≥4	81.8 [18/22] (59.7, 94.8)	96.8 [30/31] (83.3, 99.9)	90.6 [48/53] (79.3, 96.9)	94.7 [18/19] (74.0, 99.9)	88.2 [30/34] (72.5, 96.7)	
R5						0.873 (0.780, 0.967)
VI-RADS ≥3	100.0 [22/22] (78.1, 100.0)	38.7 [12/31] (21.8, 57.8)	64.2 [34/53] (49.8, 76.9)	53.7 [22/41] (37.4, 69.3)	100.0 [12/12] (64.0, 100.0)	
VI-RADS ≥4	95.5 [21/22] (77.2, 99.9)	77.4 [24/31] (58.9, 90.4)	84.9 [45/53] (72.4, 93.3)	75.0 [21/28] (55.1, 89.3)	96.0 [24/25] (79.6, 99.9)	
R6						0.862 (0.761, 0.963)
VI-RADS ≥3	90.9 [20/22] (70.8, 98.9)	51.6 [16/31] (33.1, 69.8)	67.9 [36/53] (53.7, 80.1)	57.1 [20/35] (39.4, 73.7)	88.9 [16/18] (65.3, 98.6)	
VI-RADS ≥4	77.3 [17/22] (54.6, 92.2)	80.6 [25/31] (62.5, 92.5)	79.2 [42/53] (65.9, 89.2)	73.9 [17/23] (51.6, 89.8)	83.3 [25/30] (65.3, 94.4)	
Diagnostic model	0.864 [19/22] (0.651, 0.971)	0.806 [25/31] (0.625, 0.925)	0.830 [44/53] (0.702, 0.919)	0.760 [19/25] (0.549, 0.906)	0.893 [25/28] (0.718, 0.977)	0.872 (0.777, 0.968)

Data in parentheses are 95 % confidence intervals.

Data in brackets are raw data.

VI-RADS: Vesical Imaging Reporting and Data System.

PPV: Positive predictive value.

NPV: Negative predictive value.

AUC: Area under the curve.

### 3.5 Diagnostic performance of model for semi-automated diagnosis

**Fig. 6:**
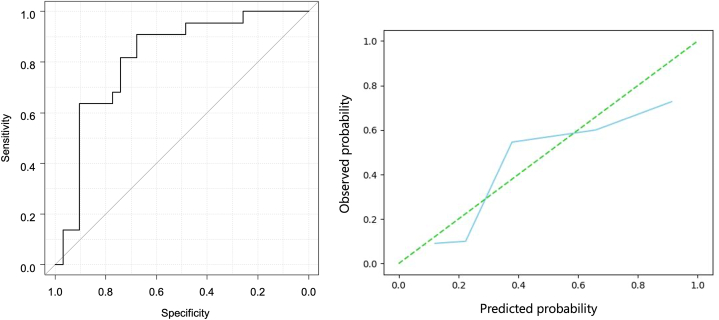
Receiver operating characteristic (ROC) curve and calibration plot of the Vision Transformer-based semi-automatic diagnosis model for the external test dataset.

The segmentation model trained using the training dataset with nnU-Net automatically detected BC in 48/53 cases for the external test dataset (Fig. S2). Four of the five bladder tumors that were not automatically detected were extremely small (4–10 mm). The other tumor was MIBC, that did not exhibit a high signal intensity on DWI. In these cases, manual ROIs were used as the input data for the diagnostic model. The final ViT-based model using these ROIs showed AUC, sensitivity, specificity, accuracy, positive predictive value, and negative predictive values of 0.815 (95 % CI:0.696, 0.935), 0.909 (20/22) (95 % CI: 0.708, 0.989), 0.677 (21/31) (95 % CI:0.486, 0.833), 0.774 [41/53] (95 % CI:0.638, 0.877), 0.667 (20/30) (95 % CI:0.472, 0.827), and 0.913 (21/23) (95 % CI:0.720, 0.989), respectively, for the external test dataset. Fig. 6 shows the ROC curve and calibration plot for the semi-automated diagnostic model. Table S2b presents the confusion matrix of the semi-automated diagnostic model.

## 4 Discussion

We developed an automated model to diagnose MIBC by integrating automatic segmentation and diagnostic models. Our model showed high diagnostic performance with high sensitivity and good calibration, which could allow for automated screening and risk stratification of muscle invasion in BC. This could reduce the efforts of radiologists in preoperative MRI evaluation. In addition, the ViT-based model outperformed the CNN-based models, demonstrating the usefulness of ViT in medical image analysis.

The diagnostic performance of our diagnostic model was comparable to that of well-trained junior radiologists. Moreover, no previous study has reported a diagnostic performance and versatility comparable to those of our model using an appropriate external test dataset. Li et al. reported two deep-learning models for diagnosing MIBC [12,14]. They used a CNN-based model to detect MIBC and achieved a high diagnostic performance (AUCs, 0.932 and 0.861) on an external test dataset. However, a non-negligible risk of overestimating the diagnostic performance of their model could exist owing to selection bias associated with the skewed T-stage distribution in their external test dataset. The test dataset included the data of 28 (Ta:7, T1:0, T2 ≤: 21, MIBC/NMIBC = 21/7) and 55 (Ta:20, T1:3, T2 ≤: 32, MIBC/NMIBC = 32/23) cases. In general, the prevalence of MIBC is approximately 25 %, which is quite different from the prevalence of MIBC based on the test datasets [30]. Notably, the extremely few T1 cases in both the reports should have affected the results because it would have been easy for the developed model to differentiate between Ta and MIBC. Further, their model used T2WI as input data and required a full tumor ROI on T2WI, whereas our model only required an ROI of one slice on DWI. This simplified the proposed model and made it more practical. Since T2WI generally provides anatomical information better than DWI, if both T2WI and DWI could be used as input data, the diagnostic ability of the model could be improved. However, accurate registration of T2WI and DWI would be a challenge due to changes in the urine volume during image acquisition. Therefore, we focused on DWI, the dominant sequence of VI-RADS, as our input data based on the assumption that DWI (b = 0 s/mm^2^) and ADC map, which are easy to register with DWI (b = 1000 s/mm^2^), and also contain anatomical information to some extent.

Our study demonstrates that the ViT-based model outperforms CNN-based models in terms of diagnostic performance. ViT has demonstrated state-of-the-art performance on various vision tasks including image classification, object detection, and semantic segmentation [15,17,31,32]. ViT is also gaining attention in medical image analysis, and some reports have indicated its potential to replace CNNs [33]. ViT has less inductive bias than CNN, and requires large datasets to outperform CNN [15]. This makes it difficult to apply ViT to medical image analysis, where large amounts of data are generally difficult to obtain. However, transfer learning or self-supervised pretraining can reportedly help overcome this difficulty [33]. In this study, we achieved a high diagnostic performance with relatively small datasets using a ViT-based model pretrained on ImageNet [23].

Our semi-automated diagnostic model also performs automated segmentation followed by automated diagnosis. In a previous study that attempted automated diagnosis, MIBC was diagnosed using a CNN-based model with automated segmentation of the BC and bladder on T2WI. The diagnostic performance of their model on the external test dataset was significantly lower than that of our model (AUC = 0.628 vs. 0.815) [13]. Our automatic segmentation model was unable to detect very small tumors, tumors, or parts of tumors that did not show a distinctly high signal intensity on DWI. Very small tumors are less likely to be MIBC. Therefore, it is not a major concern even if they cannot be detected in diagnosing MIBC. Actually, tumors with sizes of <10 mm had a VI-RADS score of 1 [6]. The low accuracy of the automatic segmentation of BC without clear high signal intensity on DWI may have contributed to the lower diagnostic performance of our semi-automated diagnostic model compared with our diagnosis model using manual ROIs. Although the addition of T2WI to DWI for automatic BC segmentation can improve the segmentation performance, accurate registration of T2WI and DWI would be challenging as mentioned above. However, if T2WI can be used as input data with non-rigid registration, the segmentation and diagnostic performance of semi-automated diagnostic models may be further improved.

Nonetheless, our study had several limitations. First, our study was retrospective and had a small sample size for deep learning. Incorporating a greater number of cases into the training dataset may improve the diagnostic performance of the model. As the diagnostic performance of the model approaches that of senior radiologists, the clinical utility of the model could be further enhanced. Furthermore, despite the validation of our model using multivendor MRI data from an external institution, it is imperative to conduct additional validation with a more extensive prospective cohort. Second, VI-RADS scoring for the external test dataset was performed using biparametric MRI, which included T2WI and DWI and not dynamic contrast-enhanced (DCE) images. Although multiparametric MRI is recommended for conventional VI-RADS scoring, the dominant sequence in VI-RADS scoring is DWI, and DCE images play an auxiliary role when DWI are difficult to diagnose due to strong artifact. Moreover, recent reports have demonstrated biparametric protocol to have a diagnostic accuracy comparable to that of the standard multiparametric protocol [[34], [35], [36]]. Besides, our test dataset excluded patients with strong artifacts. Therefore, it is unlikely that the VI-RADS scoring using biparametric MRI affected the diagnostic performance of a radiologist. Third, this study used TURBT results as the gold standard, which carries the risk of underdiagnosing muscle invasion. However, by including only cases staged according to the European Association of Urology guidelines, we believe we have minimized the likelihood of underdiagnosing muscle invasion. Fourth, although our model achieved higher diagnostic performance compared to the previous reports, it did not reach that of the senior radiologists. One reason could be that we used a single slice of BC as the input data for the diagnostic model. While this will reduce the effort required when applying the model in actual clinical practice, there is a potential for improved diagnostic performance by inputting the ROI of the entire tumor. We believe further investigation is necessary regarding this aspect in future studies.

In conclusion, we developed a deep learning model that automatically diagnoses MIBC with high accuracy and enables automated risk stratification of BC muscle invasion. Additionally, we have demonstrated the utility of ViT, highlighting its significance in deep learning research for medical image analysis.

## Funding

This work was supported by JSPS KAKENHI Grant Number JP20K16780 and 23K14866.

## Data availability statement

The data from our study has not been uploaded to a publicly accessible repository. However, it can be provided upon reasonable request.

## CRediT authorship contribution statement

**Yasuhisa Kurata:** Writing – review & editing, Writing – original draft, Visualization, Validation, Software, Resources, Project administration, Methodology, Investigation, Funding acquisition, Formal analysis, Data curation, Conceptualization. **Mizuho Nishio:** Writing – review & editing, Writing – original draft, Validation, Supervision, Software, Methodology, Formal analysis. **Yusaku Moribata:** Writing – review & editing, Writing – original draft, Validation, Data curation. **Satoshi Otani:** Writing – review & editing, Writing – original draft, Visualization, Validation, Data curation. **Yuki Himoto:** Writing – review & editing, Writing – original draft, Validation, Methodology, Investigation. **Satoru Takahashi:** Writing – review & editing, Writing – original draft, Validation, Investigation, Conceptualization. **Jiro Kusakabe:** Writing – review & editing, Writing – original draft, Validation, Methodology, Formal analysis. **Ryota Okura:** Writing – review & editing, Writing – original draft, Validation, Investigation. **Marina Shimizu:** Writing – review & editing, Writing – original draft, Validation, Investigation. **Keisuke Hidaka:** Writing – review & editing, Writing – original draft, Validation, Investigation. **Naoko Nishio:** Writing – review & editing, Writing – original draft, Data curation. **Akihiko Furuta:** Writing – review & editing, Writing – original draft, Supervision, Data curation. **Aki Kido:** Writing – review & editing, Writing – original draft, Validation, Supervision. **Kimihiko Masui:** Writing – review & editing, Writing – original draft, Validation, Supervision, Data curation. **Hiroyuki Onishi:** Writing – review & editing, Writing – original draft, Supervision, Data curation. **Takehiko Segawa:** Writing – review & editing, Writing – original draft, Supervision, Data curation. **Takashi Kobayashi:** Writing – review & editing, Writing – original draft, Supervision. **Yuji Nakamoto:** Writing – review & editing, Writing – original draft, Validation, Supervision.

## Declaration of competing interest

The authors declare the following financial interests/personal relationships which may be considered as potential competing interests: Yasuhisa Kurata reports financial support was provided by 10.13039/501100001691Japan Society for the Promotion of Science. If there are other authors, they declare that they have no known competing financial interests or personal relationships that could have appeared to influence the work reported in this paper.

## Figures and Tables

**figs1:**
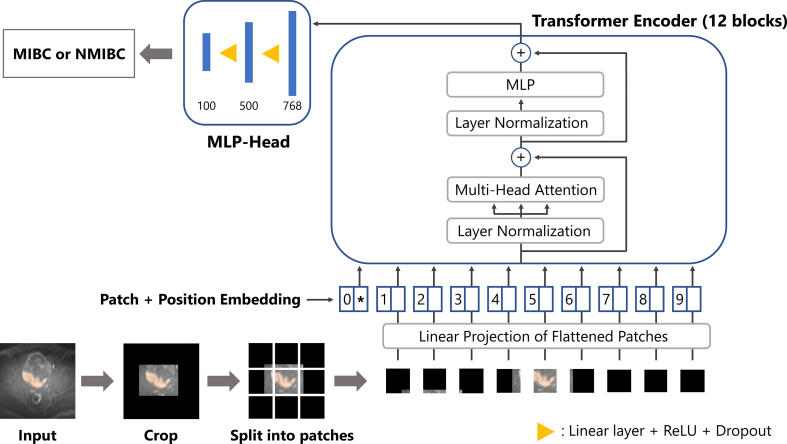


**figs2:**