# Supplementary material for: Development of deep learning model for diagnosing muscle-invasive bladder cancer on MRI with vision transformer
Source: Heliyon. 2024 Aug 10;10(16):e36144. doi: 10.1016/j.heliyon.2024.e36144 (PMC11381713; doi:10.1016/j.heliyon.2024.e36144)
Supplement: Multimedia component 1 [file mmc1.docx]

**Supplementary Materials**

**Appendix**

**(A) Image Acquisition**

Preoperative MR images were acquired using a 1.5 Tesla or 3 Tesla scanner with a phased-array coil. The following MR scanners were used: Skyra, Prisma, and Avanto (Siemens Healthcare) at Institution 1; Achieva, Ingenia, Intera (Philips Healthcare), and SIGNA EXCITE (GE Healthcare) at Institution 2; and Skyra, Symphony (Siemens Healthcare), and Achieva (Philips Healthcare) at Institution 3. The imaging protocol consisted of axial T2-weighted imaging (T2WI), axial T1-weighted imaging (T1WI), and axial diffusion-weighted imaging (DWI). Apparent diffusion coefficient (ADC) maps were automatically constructed using a mono-exponential decay model with all b-values. The MRI acquisition protocols are summarized in Table S1.

**(B) Image Preprocessing**

The MR signal intensities (SIs) were normalized using the following equation (1):

normalized_SI *=*$\frac{SI - mean\_SI}{6 \times SD\_SI}$ (1)

where mean_SI and SD_SI denote the mean and standard deviation (SD) of the SI of the images, respectively. The axial DWI and ADC maps were resized to 128 × 128 pixels outside the rectangular areas containing the segmented BC, and five pixels were filled with 0. We used three-sequence MR images (DWI of b=0 and 800 or 1000 s/mm^2^, ADC map) with the largest cross-section of the BC as the input data.

**(C) Model Development**

Our model was developed using PyTorch (version 1.11.0) and PyTorch Lightning (version 1.5.10) on a Linux workstation (Ubuntu version 20.04) with NVIDIA GeForce RTX3090, RTX4090, and Quadro RTX8000 graphics processing units with 24, 24, and 48 GB of memory, respectively.

The structure of the Vision Transformer-based diagnosis model is shown in Figure S1. A binary cross-entropy loss with logit loss was used as the loss function. In training the diagnosis model, we used the following parameters: dropout: 0.3, 0.35, 0.4, 0.45, and 0.5; learning rate: 0.001 and 0.0015; translate: 0.3 and 0.4; scale: 0.5 and 0.6; image size = 224 × 224; epochs = 1000; base size = 32; number of trainable layers = 32; weight decay = 0.001; optimizer = Adam; learning rate scheduler = ReduceLROnPlateau (patience=30, factor=0.5); and early stopping for monitoring validation loss (patience = 150). The hyperparameters of the final diagnosis mode were as follows: dropout = 0.4, learning rate = 0.0015, translation = 0.4, and scale =0.5.

**Table S1**. MRI protocols for each institution

|  | Institution 1 |  |  |  | Institution 2 |  |  |  |
| --- | --- | --- | --- | --- | --- | --- | --- | --- |
| Scanner | Skyra | Prisma | Avanto |  | Achieva | Ingenia | Intera | SIGNA EXCITE |
| Vendor | Siemens | Siemens | Siemens |  | Philips | Philips | Philips | GE |
| Patients | 61 | 19 | 4 |  | 31 | 26 | 27 | 2 |
| Magnetic field strength (T) | 3.0 | 3.0 | 1.5 |  | 1.5 | 3.0 | 1.5 | 1.5 |
| b-value (s/mm^2^) | 0, 100, 500, 1000 | 0, 100, 500, 1000 | 0, 500, 1000 |  | 0, 1000 | 0, 1000 | 0, 1000 | 0, 1000 |
| TR (ms) | 3200 | 3200 | 3900 |  | 2600 | 4000 | 2600 | 4250 |
| TE (ms) | 71 | 46 | 93 |  | 62 | 67 | 71 | 70 |
| Slice thickness (mm) | 4 | 4 | 5 |  | 4 | 4 | 3 | 5 |
| Gap (mm) | 1 | 1 | 1 |  | 0.4 | 0.4 | 0.3 | 0 |
| Matrix | 128×100 | 128×100 | 128×96 |  | 110×80 | 128×112 | 110×80 | 192×128 |
| FOV (mm) | 320 × 250 | 320 × 250 | 320 × 320 |  | 300 × 300 | 350 × 350 | 300 × 300 | 320 × 250 |

|  | Institution 3 |  |  |
| --- | --- | --- | --- |
| Scanner | Skyra | Symphony | Achieva |
| Vendor | Siemens | Siemens | Philips |
| Patients | 18 | 3 | 32 |
| Magnetic field strength (T) | 3.0 | 1.5 | 1.5 |
| b-value (s/mm^2^) | 0, 1000 | 0, 1000 | 0, 800, or 1000 |
| TR (ms) | 4500–5800 | 3500 | 1750–4750 |
| TE (ms) | 60–85 | 97–123 | 75–116 |
| Slice thickness (mm) | 4–5 | 4 | 3–5 |
| Gap (mm) | 1 | 1-2 | 1 |
| Matrix | 256×256 | 160×256 | 256×256 |
| FOV (mm) | 240 × 240 | 237 × 380 | 330 × 330 |

TR = repetition time

TE = echo time

FOV = field of view

**Table S2.** Confusion matrices of the diagnostic models for the external test dataset.

(a) Confusion matrix of the diagnostic model with a manual region of interest

| prediction | NMIBC | MIBC |  |
| --- | --- | --- | --- |
| negative | 25 | 3 | 28 |
| positive | 6 | 19 | 25 |
| Sum | 31 | 22 | 53 |

(b) Confusion matrix of the semi-automatic diagnostic model

| prediction | NMIBC | MIBC |  |
| --- | --- | --- | --- |
| negative | 21 | 2 | 23 |
| positive | 10 | 20 | 30 |
| Sum | 31 | 22 | 53 |

MIBC: Muscle-invasive bladder cancer

NMIBC: Non-muscle invasive bladder cancer

**Table S3.** Interobserver agreement of VI-RADS scores by radiologists

| Reader | R1 | R2 | R3 | R4 | R5 |
| --- | --- | --- | --- | --- | --- |
| R2 | 0.86 (0.71, 1.00) |  |  |  |  |
| R3 | 0.81 (0.64, 0.98) | 0.90 (0.83, 0.97) |  |  |  |
| R4 | 0.82 (0.72, 0.92) | 0.86 (0.78, 0.94) | 0.82 (0.73, 0.91) |  |  |
| R5 | 0.69 (0.54, 0.84) | 0.61 (0.41, 0.82) | 0.65 (0.44, 0.85) | 0.68 (0.54, 0.83) |  |
| R6 | 0.82 (0.72. 0.92) | 0.77 (0.62, 0.91) | 0.74 (0.57, 0.90) | 0.71 (0.58, 0.84) | 0.78 (0.65, 0.91) |

Data are presented as weighted kappa value (95% confidence interval).

VI-RADS: Vesical Imaging Reporting and Data System

**Supplementary figure legends**

**Figure S1.** The structure of the Vision Transformer-based diagnostic model.

A multilayer perceptron (MLP)-head composed of three layers is added to the transformer encoder. For clarity, the input image was divided into 3 × 3 patches in the figure; however, in this study, it was divided into 14 × 14 patches.

MIBC: Muscle-invasive bladder cancer; MIBC: Non-muscle invasive bladder cancer; MLP: Multilayer perceptron; ReLU: Rectified Linear Unit.

**Figure S2.** Two representative results of automatic segmentation of BC using the external test dataset.

Left to right: Diffusion-weighted images (b = 0 s/mm^2^), diffusion-weighted images (b = 1000 s/mm^2^), apparent diffusion coefficient map, automatically segmented and manually segmented regions of interest of BC.

(A) Female patient in her 70s with NMIBC. Pelvic MRI revealed an exophytic tumor with a stalk on the left lateral wall of the bladder. The segmentation model almost perfectly segmented the tumors. (B) Male patient in his 70s with MIBC. Pelvic MRI revealed a slightly exophytic tumor on the posterior wall of the bladder. The tumor did not show a distinctly high signal intensity on diffusion-weighted images (b = 1000 s/mm^2^). The segmentation model failed to detect BC.

BC: Bladder cancer; MIBC: Muscle-invasive bladder cancer; NMIBC: Non-muscle invasive bladder cancer.
